# Genomic characterization of *Enterobacter* sp. *PGRG2* and *Achromobacter insolitus PGRG5*: bacterial strains isolated from soil present near electronics manufacture industry for heavy metal remediation

**DOI:** 10.1128/mra.00617-24

**Published:** 2024-08-20

**Authors:** Ritika Garg, Shweta Dang, Pammi Gauba

**Affiliations:** 1 Department of Biotechnology, Jaypee Institute of Information Technology, Noida, Uttar Pradesh, India; University of Maryland School of Medicine, Baltimore, Maryland, USA

**Keywords:** Gram-negative bacteria

## Abstract

This article reports the whole-genome sequence of PGRG5 and the draft genome sequence of PGRG2. These strains were isolated from electronic waste contaminated soil. According to microbial identification, strain PGRG2 was identified as *Enterobacter* sp. (unclassified) with a size of ~4.4 Mbp and PGRG5 as *Achromobacter insolitus* with a size of ~6.2 Mbp.

## ANNOUNCEMENT

Heavy metals are non-biodegradable in nature and can persist in soil for thousands of years (1). Therefore, in order to make soil healthy, there is a need to clean this contamination from the soil. For this, we have been exploring the efficacy of soil microbes for the remediation of heavy metals like cadmium and lead from the contaminated soil.

Soil samples from the National Capital Region of India were collected under aseptic conditions. The samples were serially diluted in distilled water and plated on nutrient agar media (yeast extract, 2 g/L; peptone, 5 g/L; sodium chloride, 5 g/L; and agar, 15 g/L). Initially, about 22 isolates were obtained. These isolates were further streaked over nutrient agar plate spiked with Pb (NO_3_)_2_ (100 mg/kg) and CdCl_2_·H_2_O (100 mg/kg), separately. The plates were incubated for a week at 37°C. Based on these results, four isolates were chosen for further studies. On the basis of microbial identification, it was found that out of four isolates, only two were pure and identified as *Enterobacter* sp. and *Achromobacter insolitus* strain, which were further named as *Enterobacter* sp. PGRG2 and *Achromobacter insolitus* PGRG5 [PGRG2 was isolated from the soil sample of Loni Industrial Area, Ghaziabad, Uttar Pradesh, India (28.6816°N, 77.3845°E), and PGRG5 was isolated from Jhilmil Industrial Area, Jhilmil Colony, Delhi, India (28.6746°N, 77.3116°E)]. Both strains were further analyzed for whole-genome sequencing.

Whole-genome sequencing took place at Eurofins Genomics India Pvt. Ltd. (Karnataka, India). A single colony was introduced into a 50-mL nutrient broth and left to incubate overnight at 37℃ and 120 rpm within in shaking incubator. DNA extraction was carried out utilizing the commercially accessible Quick-DNA Miniprep Kit (Zymo Research) following the guidelines provided by the manufacturer. Subsequently, genomic libraries were crafted for sequencing using Illumina TruSeq Nano DNA library Prep. Kit (2). A sequencing library with paired-end reads (2 × 150 bp) was established and subsequently subjected to sequencing on the NovaSeq 6000 platform provided by Illumina (3).

A total of 6,915,857 (PGRG2) and 7,605,234 (PGRG5) raw reads were obtained (Table 1). The raw data produced were then processed to obtain clean, high-quality reads by utilizing Trimmomatic v.0.38 (4). The strains (PGRG2 and PGRG5) were initially mapped to the reference genomes using Burrows-Wheeler aligner “mem” algorithm (BWA-mem) v.0.7.17 (5) and converted and sorted into BAM file using SAMtools v.0.1.18 (6). These reference genomes, which were selected on the basis of microbial identification of the strains (reference genome for PGRG2 was *Enterobacter* sp. *SA187* (CP019113.1) and that for PGRG5 was *Achromobacter insolitus* strain *LCu2* (NZ_CP038034.1). The mapping percentage of PGRG2 and PGRG5 are 77.37% and 87.87%, respectively. A gapped consensus sequence was produced using SAMtools v.0.1.18 and BEDTools (7). Consensus sequence of 4,411,285 bp (PGRG2) and 6,428,890 bp (PGRG5) was extracted. The coverage of consensus sequence was determined using SAMtools coverage with an overall coverage of 94.29% (PGRG2) and 95.93% (PGRG5).

**TABLE 1:** Genome statistics

S. no.	Parameters	PGRG2	PGRG5
1.	Bacterial species	*Enterobacter* sp.	*Achromobacter insolitus*
2.	Isolates	PGRG2	PGRG5
3.	Accession no.	CP127837	CP129898
4.	SRA accession no.	SRR25007833	SRR25376424
5.	BioProject no.	PRJNA981674	PRJNA979826
6.	No. of reads	6,915,857	7,605,234
7.	No. of contigs	229	186
8.	Genome assembly size (bp)	4,411,285	6,428,890
9.	N_50_ contig size (bp)	48,800	136,100
10.	No. of coding sequences (CDSs)	3,801	5,689
11.	G + C content (%)	56.5	65.5
12.	No. of rRNAs	22	13
13.	No. of tRNAs	84	56

For the variant calling, SAMtools was used to identify the SNPs and InDels from the sorted BAM file of the mapping. The variants were filtered with 25% threshold, and this was annotated using BEDTools. The *de novo* assembly was made with the help of SPAdes v.3.7 (8) using default parameters, and contig annotation was carried out with Prokka v.1.11 (9). Default parameters were set for all software unless specified otherwise. The metrics were assembled using the Prokaryotic Genome Annotation Pipeline v.6.0 by National Center for Biotechnology Information (10).

## Data Availability

The genome sequence of these strains has been deposited in GenBank under the accession number listed in Table 1. Both the assembly and raw reads are available at DDBJ/ENA/GenBank under the BioProject numbers listed in Table 1.
